# The neurobiology of pain and facial movements in rodents: Clinical applications and current research

**DOI:** 10.3389/fvets.2022.1016720

**Published:** 2022-09-29

**Authors:** Adriana Domínguez-Oliva, Daniel Mota-Rojas, Ismael Hernández-Avalos, Patricia Mora-Medina, Adriana Olmos-Hernández, Antonio Verduzco-Mendoza, Alejandro Casas-Alvarado, Alexandra L. Whittaker

**Affiliations:** ^1^Master in Science Program “Maestría en Ciencias Agropecuarias”, Universidad Autónoma Metropolitana, Mexico City, Mexico; ^2^Neurophysiology, Behavior and Animal Welfare Assesment, DPAA, Universidad Autónoma Metropolitana, Mexico City, Mexico; ^3^Facultad de Estudios Superiores Cuautitlán, Universidad Nacional Autónoma de México, Cuautitlán Izcalli, Mexico; ^4^Division of Biotechnology-Bioterio and Experimental Surgery, Instituto Nacional de Rehabilitación Luis Guillermo Ibarra Ibarra, Mexico City, Mexico; ^5^School of Animal and Veterinary Sciences, The University of Adelaide, Roseworthy, SA, Australia

**Keywords:** rodents, nociception, nociceptive pathway, Rat Grimace Scale, facial action units

## Abstract

One of the most controversial aspects of the use of animals in science is the production of pain. Pain is a central ethical concern. The activation of neural pathways involved in the pain response has physiological, endocrine, and behavioral consequences, that can affect both the health and welfare of the animals, as well as the validity of research. The strategy to prevent these consequences requires understanding of the nociception process, pain itself, and how assessment can be performed using validated, non-invasive methods. The study of facial expressions related to pain has undergone considerable study with the finding that certain movements of the facial muscles (called facial action units) are associated with the presence and intensity of pain. This review, focused on rodents, discusses the neurobiology of facial expressions, clinical applications, and current research designed to better understand pain and the nociceptive pathway as a strategy for implementing refinement in biomedical research.

## Introduction

Today, pain and its repercussions constitute one of the most controversial topics in animal research. Refinement of experimental procedures involving animals is a core principle of biomedical research. As such, techniques to assess and therefore enable pain mitigation are essential (1–3). Given the widespread use of rats and mice in research, with them representing 98% of all species employed in the US and Japan (4, 5) as well as the marked increase in their use since the 20th century (6), this topic is of particular relevance to these species.

When animals experience pain during an experimental protocol, a cascade of physiological, hormonal, biochemical, and behavioral alterations are triggered, with the aim of protecting them from the harmful stimulus. However, when the duration or intensity of pain exceeds the animal's modulating capacity, homeostasis is interrupted, and persistent pain can cause consequences like hyperalgesia and sensitization (7). To avoid these adverse effects, the ideal approach is to prevent pain from occurring by providing pre-emptive analgesia. However, this is not always possible due to requirements of the animal model, or because analgesics only provide partial coverage. In these situations, opportunely identifying and evaluating pain *via* the use of appropriate tools, that then allow decision-making regarding analgesic treatments or humane endpoint implementation, is the next best scenario. In rodents the challenge of pain identification is made even greater since as prey species, behavioral signs of pain are subtle, requiring keen observation and species-specific knowledge (8).

Current research into pain recognition in veterinary medicine has led to the development and application of non-invasive tools that can assist in quantifying pain in rodents. These tools include the use of pain scales and identification of pain-specific behaviors (9). Since Darwin's (10) pioneering publications on the linkage between emotions and facial expressions of animals, the study of these phenomena has evolved to standardize their use by codifying the facial expressions associated with pain using instruments called “grimace scales.” The scales validated for laboratory mice and rats use four-five facial action units [orbital adjustment, cheek, and nose bulge (combined in rats), and the position of the ears and whiskers] that represent specific movements of muscle groups that are attributed to pain (11). These are scored on a scale from 0 to 2 based on their deviation from their basal position, to arrive at a combined score thought to indicate pain intensity (12).

The objective of this review is to discuss the theoretical bases and recent scientific advances related to the pain that laboratory rodents may perceive during research protocols. Due to the importance of pain in these settings the article addresses key concepts and characteristics of the phenomenon of pain with a particular focus on new understanding, explains the nociceptive pathway and the organic consequences that derive from its perception. In a unique way, it elucidates the association of pain with changes in the facial expressions of rodents, with an approach on the muscular and nervous neurobiology of facial movements. It also analyzes facial movements related to pain responses and the usefulness of scales based on facial expressions for evaluating pain as a strategy for recognizing and ultimately refining experimental procedures to prevent pain in laboratory rodents.

## Pain: Definition and function

The International Association for the Study of Pain defines pain as “an unpleasant sensory and emotional experience associated with, or similar to, that associated with potential or real tissue damage” (13), where the inability to express pain verbally does not preclude the possibility that it may be experienced (2). This updated definition is important since it recognizes the growing scientific evidence that animals experience the emotional and cognitive aspects of pain, and respond to this through their facial expressions, behavior, and structured body language.

**Figure 1:**
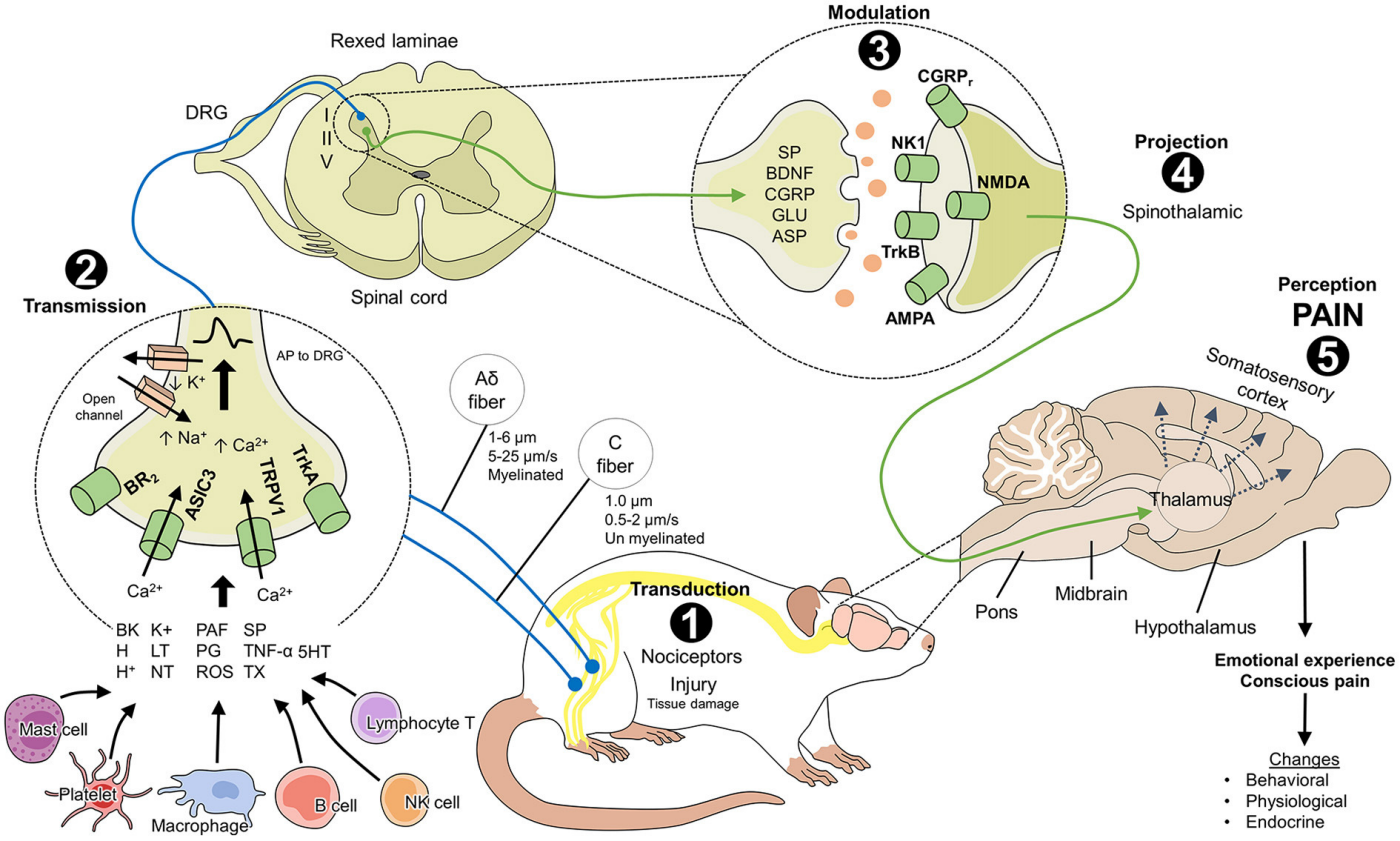
Neurobiology of pain in laboratory rats (*Rattus norvegicus*). The nociceptive pathway consists of 5 phases that are necessary to perceive pain. 1. Transduction: the transformation of a harmful stimulus into an electrical signal (or action potential) through the action of peripheral receptors (BR2, ASIC3, TRPV1, TrkA) that are activated by inflammatory mediators from mast cells, platelets, macrophages, and tissue damage. 2. Transmission: the action potentials are transmitted to the spinal cord laminae *via* first-order neurons (marked in blue) where they synapse with second-order neurons (marked in green). Laminae I, II, and V receive nociceptive input from Aδ and C fibers. 3. Modulation: the response to harmful stimuli in the inhibitory and excitatory interneurons of the spinal cord can increase or be inhibited depending on the participation of mediators such as SP, BDNF, CGRP, GLU, and ASP and their action on the membrane receptors of the postsynaptic neurons. 4. Projection: second-order neurons project the electrical signal to supraspinal centers through, for example, the spinothalamic tract, which is considered the most important for nociception. 5. Perception: once the signal reaches the higher brain centers, the thalamus connects with neurons in regions like the somatosensory cortex, where the conscious perception of pain and the consequent physiological, endocrine, and behavioral changes in rodents take place. AMPA: α-amino-3-hydroxy-5-methyl-4-isoxazole propionic acid; ASIC3, acid-sensitive ion channel 3; ASP, aspartate; BDNF, brain-derived neurotrophic factor; BK, bradykinin; BR2, bradykinin receptor 2; CGRP, calcitonin gene-related peptide; CGRPr, calcitonin gene-related peptide receptor; DRG, dorsal root ganglion; GLU, glutamate; H+, hydrogen ions; H, histamine; IL, interleukins; LT, leukotrienes; NK1, neurokinin 1 receptor; NMDA, N-methyl-D-aspartate; NT, neurotrophins; PAF, platelet-activating factor; PG, prostaglandins; ROS, free radicals; SP, substance P; TNF, tumor necrosis factor; TrkA, tropomyosin receptor kinase A; TrkB, tropomyosin receptor-related kinase B; TRPV1; vanilloid receptor type 1 transient potential; TX, thromboxanes; 5HT, serotonin.

Functionally, pain (especially acute forms) serves as an alarm and protection system that informs the organism of potential damage (14) and triggers a series of physiological and behavioral responses to prevent or reduce damage, prevent its reappearance, and promote healing (7). Nociception is the process by which a harmful stimulus is transmitted along primary afferent nerve fibers (15, 16) to higher brain structures that convert pain into a conscious experience (17, 18). This process, called the nociceptive pathway, consists of five steps (Figure 1). Activation of the nociceptive pathway and the action of the autonomic nervous system (ANS) generate a cascade of physiological, endocrine, metabolic, and behavioral responses that act to reestablish homeostasis. When pain persists, it deteriorates the health and welfare of animals due to the involvement of the sensory afferent signals and affective brain circuits that make up the nociceptive pathway (19).

## Recent findings in the study of the neurobiology of pain in rodents

### Transduction

Transduction is the conversion of harmful stimuli (thermal, mechanical, or chemical) into electrical signals or action potentials in the peripheral nerve endings of nociceptors (18). Nociceptors being nerve fibers with free nerve endings that respond to high intensity, potentially harmful stimuli (20). The transformation to electrical signals proceeds through activation of receptors or ion channels that detect different kinds of stimuli (15). The transient receptor potentials (or TRP, TRPV1-4, TRPM8, TRPA1) (18)—especially the transient receptor potential vanilloid 1 channel (TRPV1)—are known to be especially important in mammals due to their function as molecular integrators and regulators of harmful stimuli and their participation in developing nocifensive behaviors and physiological responses to pain (21, 22).

The modulation, excitation, or inhibition of these receptors has been used to understand the nociceptive pathway and develop intervention strategies during the transduction of pain. Bereiter et al. (23), for example, demonstrated that administering selective antagonists of TRPV1 reduce the expression of the receptor in rats with pain caused by severe dry eye disease induced by instilling a hypertonic saline solution and capsaicin. During processes of neuropathic pain in 3-week-old male Wistar rats, direct blocking of the TRPV1 by antagonists (AMG9810) reduced the effects of mechanical hyperalgesia in models of orofacial pain (21). This result was also observed in diabetes-mediated neuropathic pain in a study of 36 male Albino Wistar rats by Düzova et al. (24), where this type of pain increased the expression of TRPV1 in the DRG. Inhibition of transduction was achieved using antioxidants.

Antioxidants and other inflammatory mediators released by tissue damage [for example PG, nitric oxide (NO), and 5HT amongst others] (7), immune cells (e.g., IL-1β), TNF-α, nerve growth factor (NGF), neuropeptides [e.g., substance P (SP)], BDNF, and CGRP (25) go on to further activate peripheral receptors generating an enhanced response. Therefore, pathways related to these mediators represent a key target for pain relief intervention.

Inhibiting NO synthesis prevented thermal and mechanical hypersensitivity in a study of adult Wistar rats (26). Likewise, the regulation of mediators and their action on other receptors, such as TrkA/NGF, has been studied in Sprague-Dawley rat model of rectal hypersensitivity. In that work, electroacupuncture treatment reduced the expression of those channels (27). Activation of ASIC3 receptors has been shown to be associated with the pathogenesis of inflammatory bone pain (28). Studies of this kind have shown that information on harmful stimuli is transduced by the peripheral receptors whose nerve endings transmit signals to spinal structures (20).

### Transmission

The first-order nociceptive neurons, specialized in peripheral sensory activity, transmit the electrical signal from the site of a lesion to the dorsal horn of the spinal cord, passing through the dorsal ganglion to reach laminae I, II, and V (29).

Two types of nociceptors are recognized in animals: Aδ and C fibers. They are expressed in varying quantities in the dermis (12% Aδ, 30% polymodal C, and 20% mechanothermal C) (30). The myelinated, fast-conduction Aδ fibers (5–30 m/s) (20) are high-threshold mechano- and thermoreceptors (30) that have been studied as the main transmitters of pain induced by harmful cold (25–10°C) in rats of both sexes due to the latency of their action potentials (AP), of ~221 ms (31). These fibers, which are activated in nociceptive tests like the tail flick test, are present from birth in rats. Their myelinization is completed as the days pass, but their number decreases after the three first weeks of life (32).

In studies using evoked potentials techniques, stimulating the Aδ fibers is deemed the most reliable method for evaluating nociception (33). Their activation is associated with a greater frequency of pain-like behaviors, such as the paw withdrawal threshold and paw licking in rats exposed to the complete Freund Adjuvant (34). Excitability of Aδ neurons has been demonstrated to occur as a result of Ach activation of nicotinic acetylcholine receptors (35). Rodents have a large number of nicotinic acetylcholine receptors, and they thus represent a useful pain modulating target.

The C fibers are small-diameter (0.02–1.5 μm), slow-conducting (0.5–2 meters/s), polymodal unmyelinated nerve endings (30) that act by transmitting the so-called “second pain” (20, 36). Unlike the Aδ fibers, 83% of the fibers activated by harmful heat are of the C type, which have an AP latency of ~441 ms to a stimulus of 52°C (31). In newborn rats, these fibers complete their maturation within the first 3 weeks of life (37). This neuronal activation is tested in the nociceptive hot plate test commonly used in pain research (32).

The transmission of inflammatory and neuropathic pain in young female rats, and the behavioral response of spontaneous foot lifting, have been associated with models of spontaneous pain and the activation of C nociceptors due to cumulative neuroinflammation (38). The effect of pro-inflammatory substances like gamma interferon (γ) on spinal cord slices from rats has been shown to facilitate transmission from the C fibers in lamina I neurons through the action of the spinal microglia. This reaction can be attenuated by microglial inhibitors like minocycline (39). Further evidence for the role of microglia in neuropathic pain has been demonstrated using models of spinal nerve ligation in mice. These studies have shown that microglia in lamina II of the dorsal horn and the expression of receptors like P2Y12 participate in transmitting neuropathic pain. Consequently, their antagonism or complete absence in knockout mice decreases the presence of pain-related behaviors (40). Similarly, administering neuropeptides like oxytocin or orexin A and B participates in antinociception in lamina II (41).

The application of electroacupuncture to attenuate neuropathic pain in models of spared nerve injury has demonstrated that C fiber-evoked discharges are reduced by this technique and that mediators like BDNF, together with TrkB receptors, are involved in the signaling cascade of pain. They have also been suggested as mechanisms for controlling pain (42). The cumulative effect described is passed on to the ensuing phase of the nociceptive pathway, where the harmful signal is either inhibited or increased through modulation.

### Modulation

This phase involves the mechanisms that inhibit or amplify the intensity of the stimuli that reach the spinal cord (43) *via* the excitatory or inhibitory interneurons that occupy ascendent (i.e., those that project signals from the spinal cord to the encephalon) (36) or descendent pathways (i.e., those that transmit inhibitory information from higher centers). The response is then projected to the brain to begin the conscious recognition of pain (44). The signals act upon laminae of the dorsal horn of the spinal cord known as the Rexed laminae (45). The principal modulatory mechanisms are the serotoninergic pathways from the periaqueductal gray (PAG), the noradrenergic pathways in the *locus coeruleus*, those involved in production of endogenous opioids (36, 46), the endocannabinoid systems (47), and gate theory as proposed by Melzack and Wall (48).

Evidence of modulation pathways has been demonstrated widely. For example, in inflammatory pain models in rats created by causing sciatic nerve lesions the use of spinally-applied drugs which act at serotoninergic receptors has been shown to attenuate mechanical and cold-generated hyperalgesia (49). In models of neuropathic diabetic pain in rodents, Jesus et al. (50) determined that cannabidiol exerts an anti-allodynic effect also *via* the serotoninergic system by increasing the concentrations of that neurotransmitter in the spinal cord. Moreover, the participation of the different types of 5-HT receptors in modulating nociceptive responses has been studied in rats, where findings show that the 5-HT1, 5-HT2, 5-HT3, and 5-HT7 receptors are principally involved due to their expression in afferent fibers and in laminae I and II of the dorsal horn (51). In rodents with spinal cord injuries, administering agonists of 5-HT1 has also been associated with motor and postural control by inhibiting movements such as the monosynaptic stretch reflex (52). Antagonists like tropisetron or granisetron 5-HT3 (53), cyanopindolol for 5-HT1A, ketanserin for 5-HT2 (54), and acetaminophen with its analgesic, antihyperalgesic, and antinociceptive action on the 5-HT1, 5-HT2, 5-HT3, and 5-HT7 receptors, are other examples (55) of the importance of serotonergic pathways in modulation. In addition, activation of gate theory—which results in the reduction of hyperalgesia and sensitization due to the participation of Aβ axons, opioids, 5-HT, and GABA—is achieved through transcutaneous electrical nerve stimulation or spinal cord stimulation in rats with joint inflammation and non-inflammatory muscle pain (56).

Excitatory neurotransmitters like ATP, SP, and Glu, and inhibitory ones such as gamma aminobutyric acid (GABA), endogenous opioids, and monoamines (5-HT, NE) (47), play a fundamental role in modulating pain by acting on the spinal receptors NMDA, AMPA, kainate (KA), and metabotropic glutamate receptors (mGluR) (57). Studies of rats have found sex-based differences in the expression of NMDA receptors and their isoforms in the dorsal horn. While the GluN2A and GluN2D subunits are found preferentially in males, GluN2B is found in the dorsal horn of females. These findings underscore the importance of differences in nociceptive circuits (58). Additionally, the use of neurotransmitters or central modulators can reduce sensitivity to pain (44). Neuropeptide modulators have a similar effect, with neuropeptide Y (NPY) being able to reduce nociception and pain-associated behaviors, as well as allodynia and hyperalgesia in rats with lesions in the hind paws (59).

The modulation of painful stimulus influences the degree of response observed in animals, particularly pain-related behaviors. For example, Glu receptors such as mGluR2 and mGluR3 inhibit the release of neurotransmitters, and therefore have antinociceptive properties during acute and chronic pain, altering the behavioral modulation (60). Likewise, the ascending-descending pathways that modulate the response through mesolimbic mechanism in an inflammatory model of rat pain can alter behavior in the mechanical paw withdrawal or open-field test, and depend of serotoninergic and noradrenergic activity (61).

In summary, amplification or inhibition of electrical signals occurs in the spinal cord, and that response is then projected to the brain to begin the conscious recognition of pain (44).

### Projection

The projection of the nociceptive signal from the spinal neurons to supraspinal centers in the brain stem, thalamus, reticular formation, and PAG (47) occurs through the ascendent pathways in the white matter of the spinal cord. These pathways have been classified as: spinothalamic, spinoreticular, spinomesencephalic, trigeminothalamic, spinoparabrachial, and spinoparabrachial-hypothalamic (62). The spinothalamic tract is considered the primary nociceptive pathway (63). Its activation through tonic and/or burst electrical stimulation stimulates the brain areas that process the discriminative aspect of pain and the ones responsible for its cognitive, motivational, and emotional components (64). In rodents, spinal cord lesions and injuries to this tract are associated with neuropathic pain in adult Sprague-Dawley rats (65) and with the effects of mechanical allodynia and motor deficiencies that can be reduced by administering neuroprotective substances like steroids (66). Alterations of the spinothalamic and cerebral projection neurons also generate maladaptation and hyperexcitability due to the absence of modulating pathways between the fibers of the dorsal horn and the thalamus (67). Finally, the neuroendocrine response that derives from activation of the spinothalamic tract after a lesion differs between females and males, causing variations between the central mechanisms that process emotions, pain, and the ensuing phase of nociception: perception (68).

### Perception

In this final level, the primary somatosensory cortex (S1) receives projections from the thalamus (69). It is responsible for the processing, integration, and conscious experience of pain (36). EEG studies have demonstrated that when the hind paws of male Sprague-Dale rats are exposed to repetitive harmful stimuli, the S1 and anterior cingulate cortex register electrical activity during such induced painful and spontaneous pain-like events (70). As a result of this anatomical destination for projections, alterations to perception can be studied using the functional magnetic resonance technique (fMRI) due to remodeling of the somatosensory area as a result of induced neuroplasticity (71).

Since S1 neurocortical circuits participate in pain perception, dynamic neuronal oscillations (alpha, beta, and gamma) help to determine brain activity after noxious stimuli (72). Particularly, gamma oscillations are associated with acute and chronic pain, and the intensity or pain relief depends on its activation or blockage (73, 74). The induction of gamma oscillations in S1 has been shown to increase nociceptive sensitivity and the induction of aversive behaviors with the participation of the serotoninergic pathways (75). These results are similar to those reported by Peng et al. (76), who determined that gamma band oscillations are the only ones that correlate with such pain-related behaviors as flinching, withdrawal, and licking of the zone exposed to nociceptive stimuli in male rats.

Current techniques such as *in vivo* Ca2+ imagining of the anterior cingulate cortex (ACC), another structure involved in pain perception (77), are used in acute and chronic pain models in mice. In Zhao et al.'s work (78), noxious pressure stimulation evoked and enhanced the electrical activity of the ACC layer 5 neurons in mice with sciatic nerve injury. During acute pain, a rise in the somatic Ca2+ transients was reported. This response was associated with the intensity of the stimulus and induced the paw withdrawal reflex. In chronic pain states, the Ca2+ activity was 2-fold higher than in sham animals, and mice with nerve injury had mechanical allodynia. Allodynia is also present in mice models of dry eye disease. The modulation of nociceptors activity, such as TRPM8 in these cases can be evaluated using an electrophysiological multi-unit extracellular recording. Fakih et al. found that the topical administration of TRPM8 antagonist (M8-B) decreased the activity of the ciliary nerves, serving as a local antalgic agent for ocular pain (79).

The emotional and affective processing of pain can be evaluated through optogenetics. Fiber photometry-based Ca2+ imaging revealed that dopamine, an important neuromodulator of pain-related behavior in mammals, increases the activity of the media prefrontal cortex neurons in the ventrolateral PAG and modulates responses to neuropathic pain by descending pain pathways (80). Likewise, fiber photometry showed that pathways involving glutamatergic neurons in the basolateral amygdala, insular cortex, and the mediodorsal thalamic nucleus upon inflammatory pain activate and modulate aversive responses to pain in mice (81). MRI and neural connectivity with ultra-high-field in rat models of knee chronic pain showed connectivity between ACC and subcortical structures, as well as a suppression of burrowing, a behavior associated with the presence of pain (82).

During pain perception, the reciprocal relationship between this phase and other states of affect also influences degree response. For example, pain-induced depression is observed in rats with persistent neuropathic pain (and is also reported in humans). Rats with spinal nerve transection treated with antidepressants (rosiglitazone) ameliorate depressive-like behaviors by modulating neurotransmitter levels in the hippocampus (83). In depressive states in rat models of chronic postsurgical pain, the administration of ketamine (a compound known for its analgesic and antidepressant effects), decreased proinflammatory mediators (IL-1, IL-6, and TNFα) in the hippocampus and increased BDNF levels. This led to reduced depression-like behaviors without an effect on hyperalgesia (84). Anxiety is another comorbidity associated with chronic pain, including those of neuropathic and inflammatory origin (85). The importance of recognizing emotional pain is that it can regulate the intensity of physical pain, and is related to the levels of neurotransmitters that also participate in the modulation phase (86).

To summarize, perception of pain is the process through the which the brain recognizes this phenomenon as an unpleasant sensory and emotional experience associated with nociceptive transmission that culminates with the presentation of affective, behavioral, autonomous, and motor responses as mechanisms to confront the pain and impede additional damage (47).

## Organic responses derived from pain in relation to pain assessment

At the onset of a nociceptive response, a cascade of organic and biochemical alterations activates the sympathetic-adrenal-medullary axis (SAM) or the hypothalamic-pituitary-adrenal axis (HPA), causing the secretion of glucocorticoids and catecholamines (87). These alterations in the animals' immune, biological, neurological, and physiological functions predispose them to pathologies due to immunosuppression that, in addition to affecting their health, may also have repercussions for results by altering physiological parameters such as heart rate, respiratory frequency, and blood pressure, as well as altering behavior (88). For this reason, it is key that refinement in animal pain research is identified and controlled to the greatest extent possible. A discussion of some of these organic responses follows.

An objective pain assessment method does not currently exist, in spite of a wide variety of biomarkers and behavioral methods being available that may be suggestive of pain. Therefore, in general, pain assessment requires the integration of a number of changes in physiological, biochemical, endocrine, and behavioral parameters (89), to infer a painful state. It is however clear that these changes are not exclusive indicators of pain in any animal species (90, 91).

### Physiological, endocrine, and metabolic responses

The integration and perception of pain in the CNS generates physiological responses that can include tachycardia, tachypnea, hypertension, mydriasis (92), and hyperthermia of ~1.7°C (93). These responses result from participation of the hypothalamus, and activation of the SNS and its primary axes: HPA and SAM (3). The SAM axis increases circulating catecholamine concentrations while the HPA axis contributes to the secretion of glucocorticoids (92). As a result the main endocrine change is an increase in corticosterone, although there are reports of increases of other hormones, such as the adrenocorticotropic hormone (ACTH), oxytocin, and prolactin, as well as decreases in the growth hormone (GH) (94).

Corticosterone is however most commonly used as a biomarker, and can show substantial change. In rodents, corticosterone is the major output of the HPA axis (95), due to the lack of the CYP17α enzyme (96). For example, during acute pain in rats, levels increased as high as 385% compared with glucose at 30–195%, and prolactin up to 275% (93). It is important to note that corticosterone and some other markers are useful only in cases of acute pain (97). Because corticosterone regulates diverse activities, its increase signals alterations in the metabolism of carbohydrates, proteins. and fats (98). The effect of corticosterone can be manifested as hyperglycemia and lipolysis, which can even put an organism into a diabetogenic state (99), and alter thermoregulating mechanisms (100) in both awake and surgical patients (101).

Other biomarkers that have been tested for their usefulness as indicators of pain, aside from glucose, include free fatty acids, lactic acid, ACTH, SP, beta-endorphins, and acute phase proteins. However, these biomarkers, just like corticosterone, are not pathognomonic of pain. As a result they are generally used as part of an assessment integrating a number of indices that allows us to infer that pain is being experienced by animals (92). Since the physiological response to pain is often non-specific, behavioral patterns represent an alternative avenue for the study of, and clinical recognition, of pain.

### Behavioral responses

It has been widely suggested that in prey species such as rodents, detecting pain-related behaviors from early manifestations is challenging because these animals tend to conceal signs to protect themselves (92). Weary et al. (102) consider that using these behaviors as a method for evaluating pain requires, first, distinguishing three types: (1) pain-like behaviors; (2) changes in the frequency of certain behaviors; and (3) preference behaviors.

Pain-like behaviors include vocalizations, flight responses, withdrawal of a body part, agitation, reduced mobility, repetitive grooming or licking of the injured area, back-arching, writhing, and twitching (103, 104). However, many of these responses may be unique to certain pain types, for example back arching, writhing and twitching have mainly been noted after abdominal surgery. Spontaneous pain behaviors in rats and mice, such as alteration in locomotor and gait activity, walking, stretching, or licking the injured area are considered pain indicators (9). In male rats, hind paw licking can be seen after injection of formalin (105). However, as Draxler et al. (106) state, the manifestation of the pain behavior depends on the pain model (inflammatory, postsurgical, cephalic, neuropathic, or chemotherapy-related), and analgesic therapy can reduce the frequency of behavioral changes.

A change in frequency of feeding is commonly used as a pain indicator in both acute and chronic pain (107), and can of course be reflected in body weight changes. A recent study has also investigated refeeding-induced analgesia in inflammatory pain, determining that the mechanism of this response *via* neural activities in the nucleus accumbens and anterior insula cortex may be a target target for chronic pain management (107). Measuring bodyweight is often the mainstay method used by researchers to assess pain in biomedical research as part of daily animal health checks. Talbot et al. (108) mention that a weight reduction of 20% or more is considered a humane endpoint in animal research.

Weight loss is commonly used to evaluate postoperative pain and the efficacy of analgesic drugs (e.g., meloxicam or buprenorphine). In Lewis male rats, Brennan et al. (109) determined that a reduction of more than 3% of daily weight gain could be an indicator of pain and a cut-off point to reconsider the pharmacologic treatment. This was also observed in Sprague-Dawley and Dark Agouti rats undergoing laparotomy. Regardless of the analgesic drug, all animals lost weight and reduced their food and water intake on the first postsurgical day. Nonetheless, the weight reduction was lessened in animals receiving buprenorphine (110). In rat models of diabetic neuropathy, antihyperalgesic components such as rosemary extract significantly increased body weight at the end of the study (from 231.7 ± 4.326 to 241.2 ± 4.143 g), and also reduced the progress of diabetes-induced thermal hyperalgesia (111), showing the association between antinociception and weight maintenance. However, it is non-specific, since reduced feeding may also be triggered by malaise or nausea. This means that interventions specifically targeted at pain may fail. Moreover, the assessment of feeding behavior is generally impractical unless automated home cage monitoring is used, and it can take some time for changes in weight to be apparent, rendering the latter a relatively insensitive pain assessment method (112).

Nevertheless, in oro-facial disease models where tissue damage directly impacts eating function, assessment of eating behavior, is a common and reliable method. In rats, capsaicin-induced dental pain caused a reduction in food intake (113). Another example is the evaluation of meal duration, a measure that can be used as a non-invasive method to recognize nociception in rat models of induced temporomandibular pain, where joint inflammation impairs and slows their eating patterns (114). Restoring the normal meal duration can serve as an indicator of the pharmacologic efficacy of anti-inflammatory drugs such as dexamethasone (115), or capsaicin, a compound known to eliminate C-fibers that participate in the nociceptive pathway (114). Similarly, mice with temporomandibular joint pain had decreased eating duration and frequency, an effect that was consistent with cartilage degradation, making it a reliable method for pain recognition (116). Adequate dosage of multimodal analgesic treatments with opioids and non-steroidal anti-inflammatory drugs after a surgical procedure has been shown to preserve food intake in rats undergoing implantation of epidural electrodes (117). The neuronal pathway behind these changes has been evaluated by Hogri et al. (118) in male Sprague Dawley rats, who demonstrated that stimulation of neurons in the central nucleus of the amygdala, known for its role in nociceptive integration, decreases the presentation of nocifensive behaviors and promote food intake and appetite due to analgesia. A reduction in gnawing efficiency has been used to detect oral cancer pain through use of an instrument called a dolognawmeter (119). Since gnawing is similar to chewing and uses the same masticatory muscles, this behavior is indicative of function-related pain that depends on its intensity, the sex of the animal, and analgesic therapies such as neutrophil-mediated analgesia (120).

Another pain-related behavior that is relevant in human-like diseases such as migraine is light aversion (121). Light-aversive behaviors were evaluated in rat models of induced migraine (122). In these animals, antinociceptive treatment with a multimodal neuropeptide agent reduced these behaviors and mechanical/thermal hyperalgesia (122). Events of hyperalgesia, allodynia, and photophobia, together with increased serum cortisol levels, were also observed in male Wistar rats, a response that was attenuated with the administration of ghrelin (123), a peptide that reduces the intensity of inflammatory pain through the secretion of anti-inflammatory cytokines (124). In mice, Shepherd et al. (125) reported that the color of the burrow tube influences burrowing performance, suggesting that light intensity in a lit room can induce aversion. This effect has a relation to the expression of CGRP in the medial nucleus of the cerebellum, where the stimulation of these neurons in female mice causes hypersensitivity to light (126).

There has been recent focus on the use of non-maintenance behaviors, sometimes named “luxury” behaviors, as an indicator more generally of affective state, which is usually impacted by pain. One example of a change in the frequency of a natural behavior in rats involves burrowing (17, 127). A lower frequency of burrowing has been associated with acute and chronic visceral pain (103), post-operative pain, osteoarthritis, and inflammatory (17) and neuropathic pain (128). This behavior has been also used to evaluate analgesic efficacy in rodents (129).

Nesting is another natural behavior in rodents, considered an activity of daily living or a luxury behavior (127, 130). The evaluation of the time spent in constructing the nest and its quality has been used as an indicator of pain in mice, including those in models of osteoarthritic pain. In the study by Dutta et al. (131), mice without pain and those treated with an analgesic compound (MCC22) formed more robust nests with no reduction in the functionality of the animals. Similarly, in mice undergoing vasectomies or females undergoing sham embryo transfer, administration of local analgesics (lidocaine and bupivacaine) increased nest complexity in males and females between 12 and 24 h after the surgery (132). Therefore, the reduction in nest building is considered an indicator of diminished welfare during stressful conditions such as pain (133). However, authors such as Tappe-Theodor et al. (130) mention that the interruption of this type of behavior is not specific to identifying the pain since any stimulus that disrupts the wellbeing of rodents will generate an alteration in their behavior. Likewise, one must understand the natural propensity to burrow dependant on strain, sex species and individual characteristics (134).

In this context, the pain experience could differ within the same species and between individuals due to genetic, genomic, epigenetic, environmental, and psychological factors (135, 136). This involves differences in pain sensitivity, susceptibility to painful disorders, or efficacy of analgesic drugs (137). Traits such as temperament, sociability, or anxiety, among others, also play a role in pain-related behaviors. For example, a study of neuropathic pain in mice with low sociability and high anxiety phenotypes evoked neuronal activity in the amygdala and an enhanced hypersensitivity response to nerve injury (138). In female rats with strong fear extinction, models of acute and chronic pain (arthritis and neuropathic, respectively) had fewer vocalizations, since the emotional components of pain (such as fear) can alter the perception of pain and alter analgesic response (139).

The age and sex of rodents needs to be considered in pain studies. Sex is a key contributor to differences in response to pain with now almost widespread acceptance that there are differences in pain thresholds between male and female rodents, with females having a lower pain threshold in response to a variety of nociceptive inputs (127). Inter-individual differences are a current area of research (135), where behavioral individuality could affect the intensity of response or the individual's pain threshold.

Likewise, as Mogil (140) states, there is a large interindividual variability in rodents due to genetic or heritability influences in the different strains used in biomedical research, such as the recombinant inbred CxBK mouse, the High Analgesia/Low analgesia, High Analgesic Response/Low analgesic Response, High Autotomy/Low Autototomy, and normotensive or hypertensive Wistar Kyoto rat (140). This interindividual difference has also been reported in rat models of neuropathic pain, where more than 40% of Sprague-Dawley rats do not respond to analgesic therapies such as electroacupuncture (141). Studies regarding this issue have shown that differences within the same strain can be attributed to the expression of anti-opioid peptide cholecystokinin CCK-8, a component associated with individual sensitivity (136). In murine models of anxiety, differences between mice of different strains have also been documented (BALB/c –neophobic mouse strain–, C57BL/6, and 129S2). BALB/c animals are known to be highly neophobic but quickly adapt to their environment, so their response to the stressor may be diminished. Contrarily, 129S2 presents increased avoidance behavior and a higher physiological response to stress, assessed with increased avoidance behavior. therefore, display a greater amount of anxiety behaviors. These differences were attributed to a low expression of c-Fos, a marker for neural activity in the prelimbic cortex and lateral septum, areas involved in the emotional response of animals (142). Likewise, through genetic mapping in mice, it has been shown that protein expression of the subunit β3 in the DRG contributes to pain sensitivity and strain differences between A/J and C57BL/6J (143). These few examples are the tip of the iceberg; there is clear evidence across a range of rodent stocks and strains that individual epigenetic processes are strong influencers of physiological and behavioral responses in animal models.

Another approach to pain assessment which uses behavioral observations is the use of facial expression scoring. Studies of rodents, mainly rats and mice, have identified that the degree of muscular changes in the face is associated with the intensity of pain that the animal experiences, i.e., this method is thought to identify the affective component of pain. These changes involve the position of the ears and whiskers, orbital tightening, and flattening of the nose and cheeks (17).

## Facial expressions, facial action units and the development of grimace scales

Facial expressions are the result of involuntary muscular responses to emotional stimuli creating changes in a group of facial muscles. These groups are called facial action units (FAU). Facial expressions play a role in communication that has been shown to be influenced by the social context of the emotion and the affective states of animals (144). Further, they function as a means of social exchange that allow one organism to respond to others (145). Ekman and Friesen (146) were the first to methodically study facial expressions. They developed the Facial Action Coding System (FACS) as a method for identifying the movements and positions of facial muscle groups in relation to universal emotions (147). The FACS system encompasses all anatomically possible facial movements and assigns a name to each one so they can be used in various fields, including veterinary medicine. FACS describes 44 FAU in humans, each one representing the activation of a muscle measured on a 5-point intensity scale (147, 148). Though described as an objective method of evaluation, FACS has the disadvantage that it considers only facial movements that are clearly visible, omitting more subtle changes and other facial phenomena—such as skin coloration—as well as tearing and sweating (149).

Darwin was the first author to attribute changes in behavior to emotions, including pain, to non-human animals, and to describe how facial expressions reflected them. These expressions were conceived as innate, adaptive, evolutionarily-conserved responses (10). Research on facial expressions in the past decade has analyzed observable movements of facial muscles in animals with the goal of associating them with specific events or emotions (150). Studies have focused on those FAU which are observed frequently in all animals, such as orbital tightening associated with pain (151). There has also been demonstration that facial changes are not only associated with negative emotions (described below), but also positive ones. As an example rats exposed to positive stimuli like gentle handling and tickling showed expression changes manifested as changes in the height and width of the eyes and the color (more pink) and more relaxed position of the ears (152). Likewise, facial movements associated with positive emotional states have been reported in behavioral tests such as the elevated plus-maze. Lecorps and Féron (153) found that ears were positioned in an upright and forward position in mice who openly explored their surroundings, when exposed to a novel odor, suggesting this was an indicator of emotional reactivity. In tests using palatable foods for rats, facial expression also varied suggesting an association with positive affective experiences (154). The advantage of facial expressions for evaluating pain in animals is that the method is simple and the response outwardly visible. This could then allow veterinarians and caregivers to rapidly assess painful state to allow mitigation through therapeutic administration or technique modification (151).

## The neurophysiology of facial expressions and their relation to pain

Human facial expressions have been widely studied to assess the emotional and affective experience of pain. In the case of animals, the belief that facial expressions are not under voluntary control (except for non-human primates) suggests that certain movements of the facial muscles can indicate affective states like pain and emotions (155).

**Figure 2:**
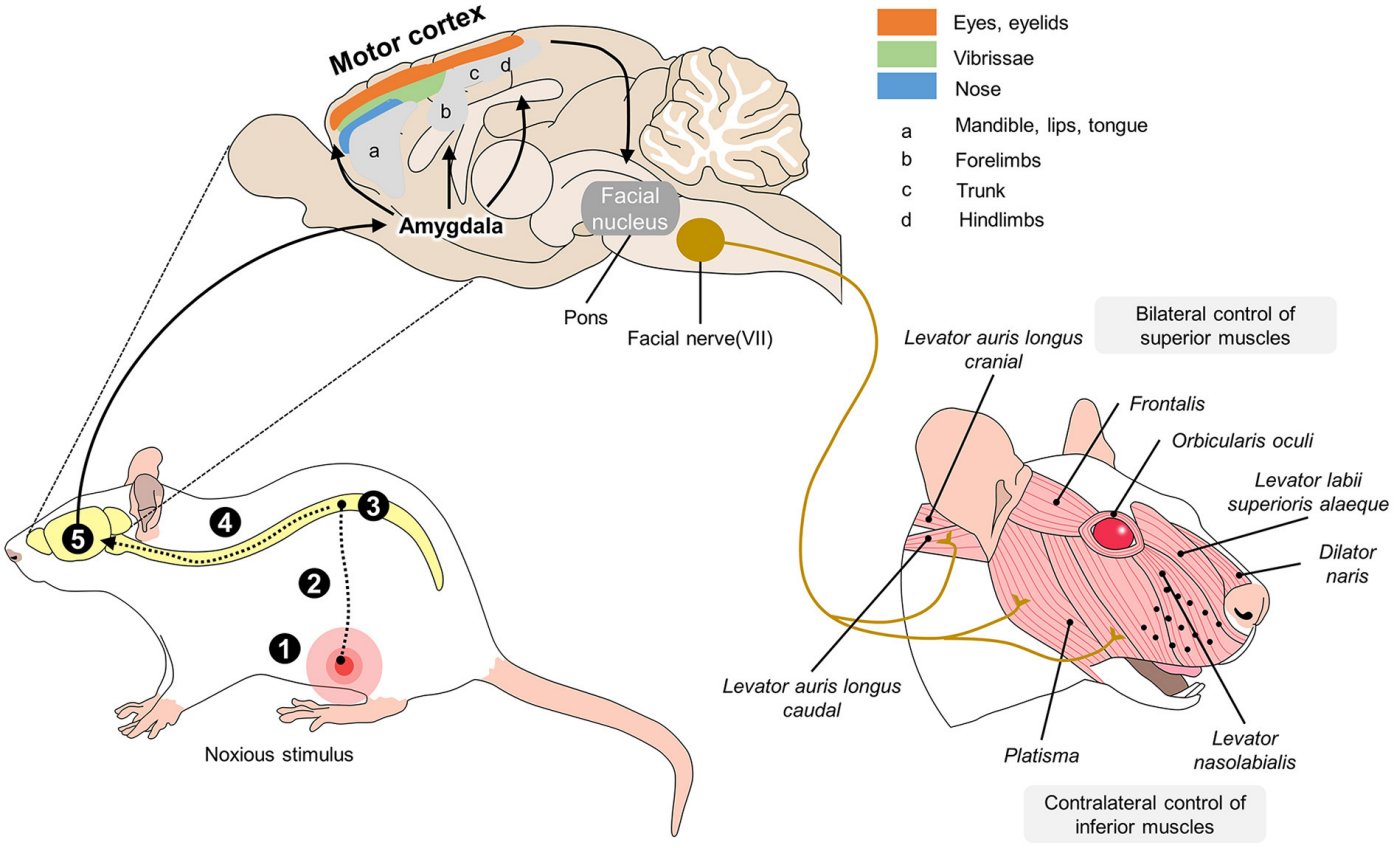
Neurobiology of facial expressions in R. norvegicus. The production of facial expressions associated with pain begins with the nociceptive pathway. When the nociceptive signal reaches the brain, the thalamus projects connections to the amygdala, the main center that initiates facial motor responses. From the amygdala, the motor cortex is activated in various areas responsible for controlling the eyes, vibrissae, nose, jaw, and pelvic and thoracic limbs. Once activated, areas of the motor cortex connect directly to the facial nucleus in the pons and, in turn, with cranial nerve VII, which innervates all the muscles involved in facial expressions. In rodents, the muscles described in the image are responsible for maintaining the position of the ears and whiskers and movements of the eyelids and nose, which are considered rat-specific facial action units (FAUs) for pain recognition. 1. transduction; 2. transmission; 3. modulation; 4. projection; 5. perception.

The manifestation of facial expressions begins with the perception of a stimulus. That stimulus does not depend only on an anatomical component, but is the result of the activity of a circuit that integrates subcortical and cortical areas like the amygdala, primary motor cortex, ventrolateral motor cortex, and supplementary motor area, as well as two dorsal motor areas of the midcingulate cortex and the motor fibers that innervate the facial muscles (144, 145). In the case of a painful insult, the nociceptive pathway's third-order neurons that project the nociceptive stimulus to the somatosensorial cortex also connect to the amygdala (for emotional responses), hypothalamus (to generate autonomous responses), and motor areas of the cerebral cortex. The latter contain the final-order motor neurons that directly innervate the facial muscle fibers according to the signal sent from the circuit of cortical motor neurons (156, 157) (Figure 2).

Based on the study of FAU and their relation to pain, researchers in veterinary medicine have developed grimace scales to score the diverse facial expressions associated with pain in various animal species (158). Langford et al. (11) were among the first to put such scales into practice using the Mouse Grimace Scale. That tool has been shown to have a precision of 72–81% for detecting signs of pain and can differentiate between sensory (abdominal contortions due to pain) and emotional responses reflected in facial expressions (11). This success has led to proposals of pain scales—grimace scales—for may domesticated species, including laboratory rats, horses and cats (155, 159). These scales enable observers to determine the absence/presence of pain and its severity, since this correlates with the intensity of the expression observed (11). In research with rats, these scales have been used to study pathologies, biological processes, and physiopathological mechanisms of pain that would be difficult to assess in humans or would raise serious ethical issues (17).

## Rodent grimace scales

**Figure 3:**
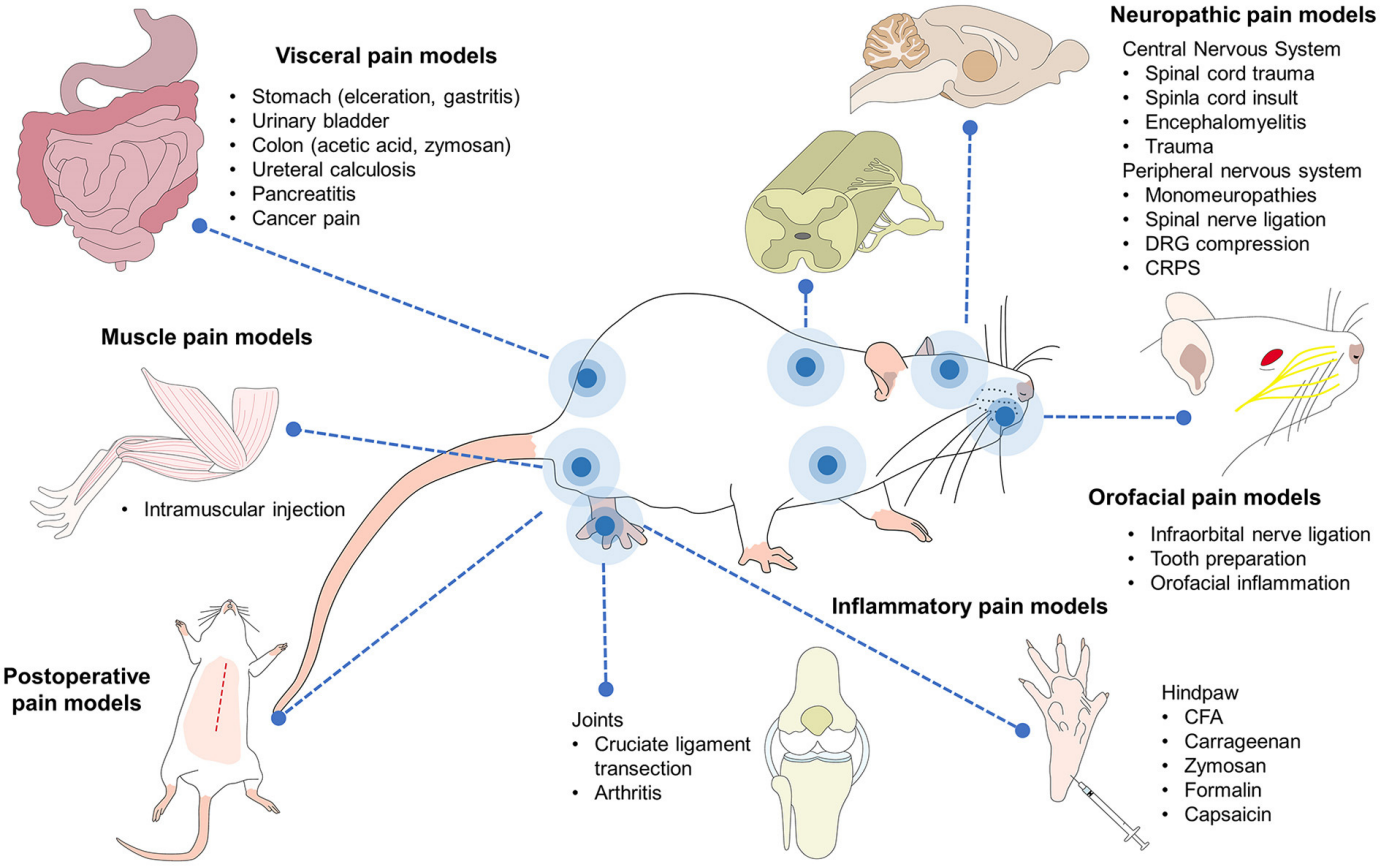
Pain models in rodents. Current research with rodent models includes several aspects of medicine and biological sciences, including pain. Several inflammatory, neuropathic, visceral, muscle-skeletal, and postoperative pain are currently studied in rodents. Orofacial, cancer, head, and burn pain are also part of the biomedical research where mice and rat play a key role in the comprehension of the physiopathology as well as the development of analgesic therapies.

The pain scales developed and now applied in veterinary medicine began with rodents due to their importance in studies of diverse pathologies for biomedical research purposes (Figure 3), which involved both spontaneous or induced pain (11). They were also derived with clinical application in mind due to the challenges in evaluating negative states like pain in rodents (160). Their development has been based on taking images of animals with and without pain by video recording or using still photos, with or without the use of software such as the “Rodent Face Finder,” which automatically selects specific frames where the face of the rodent can be seen clearly (151).

The Rat Grimace Scale (RGS) was developed by Sotocinal (161) and validated by Oliver et al. (162). It was elaborated after its counterpart for mice (the MGS) by conducting three algesiometric assays (intraplantar injection of the Freud adjuvant, intraarticular injection of kaolin-carrageenan, and laparotomy) utilizing the conventional method of digital video recording for 30 min before the injections or surgery to capture images of the “absence of pain” and 30 min afterwards to evaluate responses, obtaining some 500 images. Though based on the MGS, there are differences in the FAU of these two species. In rats, the nose and cheek area are flattened when pain is felt, so only four FAU have been designated for this species. As Di Giminiani et al. (155) reported, an FAU is designated when the same movement occurs consistently in 25–50% of observations.

The FAU used with rats are controlled by the facial nerve that innervates two muscle groups: the superficial muscles and a set of three deep muscles. The ones associated with facial expressions are the *nasolabialis* (including the *levator labii superioris* and *dilator daris* muscles), the *levator labii superioris* (superficial) and its fibers—which control the movement of the whiskers—together with the *dilator naris* (163). In mice, the motor control of the vibrissae is associated with pathways at the mesencephalic trigeminal nucleus (164). Regarding species, there are some differences that must be considered when assessing pain through facial expression. In the case of mice, naturally, there is no bulging of the nasal bridge and cheeks, a change that is noticeable when the animals experience severe pain (165). This difference influences the amount of FAU evaluated in each species (166). For example, while rats use four FAU the MGS employs five different muscular movements because bulging of the nose and cheeks is not always observed together (167).

Whilst it is recognized that there are study differences in the reported reliability of use of facial expressions to diagnose pain, and that reliability values differ based on whether a binary determination of the presence or absence of pain is sufficient, or if gradation of pain response is required. However, in spite of this variability the technique has been shown to have a validity of 81.6%, with no difference between the precision of photos taken from a frontal or profile angle, an exactitude of 76–87.5% for identifying facial expressions of pain, a sensitivity of 89.7%, and a specificity of 91.8%. Adequate training of the observers can increase the reliability of these recording to 90% (161), though some authors mention that using FACS adequately in humans requires as many as 100 h of training (148).

**Figure 4:**
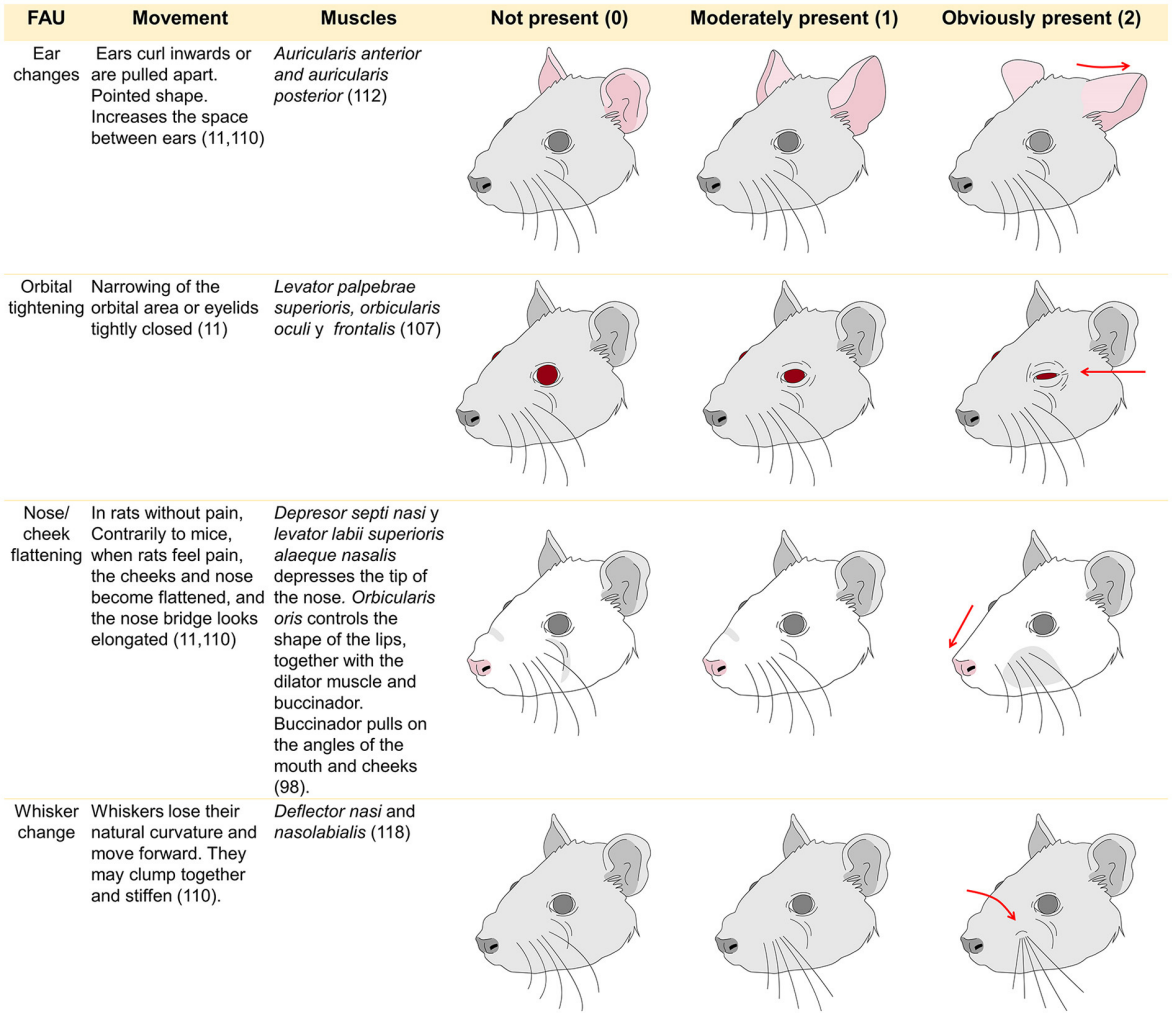
Facial action units used to evaluate facial expression in rats and their muscles involved in its control.

Pain is evaluated on a scale of 0–2 based on the degree of deviation of the FAU from expected (typical) position with FAUs being scored individually. The 0 means “not present,” 1 indicates “partially or moderately present,” and 2 denotes “markedly present.” The final value is calculated by assimilating the scores of all the FAU either through averaging or summation (147). In some species intervention thresholds, at which it is considered necessary to administer rescue analgesia, have been described. Although these values likely need further investigation and validation (162, 168). In general most studies of grimace scales in rodents have employed models where pain was expected to be momentary or acute in nature (from a few minutes to several days) and this is where the strongest evidence for their utility exists (160). However, there is limited evidence of their utility in manifestations of chronic, neuropathic, and orthopedic pain which are expected to be more chronic in nature and that can implicate pain-related stress (169). Figure 4 summarizes the four FAU used in rats as a representative species. Facial expressions have been studied using these FAU to determine degrees of pain in diverse research protocols. The FAU have been shown to be simple, non-invasive, real-time or retrospective tools for recognizing pain in rodents (11, 147, 158, 161, 163, 170).

## Clinical applications of facial expressions in distinct experimental models of pain in laboratory rodents

From a clinical perspective, facial expressions have the potential to allow implementation of timely measures to minimize the suffering of laboratory rats and other animals (171) through administration of pain relief or implementation of humane endpoints. They have also been used to re-evaluate dosing regimens in laboratory animal medicine. There are however study differences in the sensitivity of the scales for detection of pain. This may be related to individual study characteristics, or the type of pain expected to be experienced.

Leung et al. (129) used changes in facial expressions as a technique to refine simple methods of analgesia with buprenorphine and multimodal opioid analgesia with meloxicam and a control group (saline solution). After conducting an evaluation in real time using conventional videorecording, those authors concluded that both approaches can discriminate between the group that received analgesia and the one that did not, where the opioids reduce RGS scores compared to the control group and made it possible to identify pain early and quickly. In contrast, in models of chemotherapy-induced visceral pain, the RGS showed no significant differences in Dark Agouti rats compared to the disease activity index, and was not modified by use of opioid agents (172).

The MGS has been applied in craniotomy models to test the efficacy of post-surgical analgesics such as carprofen, meloxicam, and buprenorphine. In this study, MGS scores decreased in the first 24 h post-surgery (*p* < 0.001), with buprenorphine being the most effective drug in reducing scores at 8 h (*p* = 0.046) (173). During more common procedures, such as intraperitoneal administration of substances (e.g., CCl4 and oil as a control group), Erns et al. (174) reported that orbital narrowing was the most observed FAU in the MGS in mice after the intraperitoneal injection of CCl4, demonstrating that MGS can detect pain depending on the agent administered. Likewise, Heinsinger et al. (175) mentions that in murine models of cervical spinal cord injury, the nose and cheek bulge, orbital narrowing, and change of ear position are the most obvious FAU after 2 weeks of cervical contusion. This information does not only show the applicability of the grimace scales and FAU to pain recognition but is also an alternative to decide an analgesic approach for laboratory animals.

The sensitivity of RGS for communicating pain has been compared to other evaluation scales, such as the Composite Behavior Scale, which is based on body postures that denote pain; for example, writhing, arching the back, and staggering. In these cases, the scales were utilized to discriminate the analgesic effect of meloxicam and buprenorphine in a surgical model of laparotomy. Both scales showed higher scores during the 390 min of evaluation, but only the RGS scores descended when buprenorphine was administered, suggesting greater sensitivity for the study of facial expressions to distinguish between animals with or without pain (176). Those findings are similar to the report by Leung et al. (103), who compared these two scales with respect to the frequency with which the animals' behavior—in this case, burrowing activity—occurred in a model of colitis-induced acute and chronic inflammatory pain. The comparison of the scores obtained using facial expressions and the disease activity index showed that both increased during the acute and chronic phases, but that only burrowing decreased during the phase of acute pain. Their findings led the authors to conclude that the RGS can be used in cases of chronic pain, as was reported in a case of chronic pain caused by damage to the infraorbital nerve (177).

The facial expressions of rats have also been used to evaluate neuropathic pain caused by cervical radiculopathy, and have been validated for visceral, surgical, orthopedic, and inflammatory pain (178). Today, some automatic systems use computers to learn to recognize these facial changes. These systems can distinguish various facial expressions (179), but it is recommended that evaluators receive some type of training to detect changes in the FAU (180), since the study of pain and stressful events in animals used in science can be influenced by experience and the subjectivity of evaluations (181).

## Areas of opportunity regarding pain assessment and the implementation of new techniques

There is a tendency in pain assessment of rodents to use a combination of methods which might include facial expression scoring, and use of other non-invasive techniques, such as quantifying bodyweight change (3). However, there has been less focus on the use of behaviors known to indicate positive states, such as allo-grooming, and nesting (182, 183). Whilst, these may be non-specific to pain, given the linkage between pain and emotion, for example depression as a co-morbidity in chronic pain which is prevalent in human populations, this may enhance validity and reproducibility rather than detract from the specific hypothesis testing goals. It is argued therefore, that there remains an opportunity to broaden current behavioral-based assessment techniques to consider assessment of the absence/presence of natural behaviors and vocalizations (184). Provision of resources that encourage these behaviors, and training of staff in the value of them, may also be beneficial in avoiding or mitigating pain. For example, tickling techniques applied to laboratory rats imitate this species' heterospecific play behavior and have been shown to improve mental state. There does however need to be consideration of the practical implications and limitations of using some of these methods. For example, the uptake of tickling is thought to be low in facilities due to researchers' lack of time, personnel shortages, or limits imposed due to experimental design (185). Alternately, there are other assessment techniques that may, with some further development, be minimally labor intensive. For example, the analysis of ultrasonic vocalizations as an indicator of affective state is objective, sensitive to valence of affective state and can be done non-invasively. Studies report that high (~50 kHz) or low frequency (~22 kHz) vocalizations are associated to positive and negative experiences, respectively (186).

Employing environmental enrichment has also been associated with positive mental states and enhanced cognitive and learning capacities in rodents (187). Enrichment use has historically been controversial with some researchers stating that these practices need to be standardized to ensure that the studies that result are replicable and valid, and do not compromise findings or the potential for comparisons with earlier research (188). However, the proposition that standardization through reducing environmental variability is beneficial to research has been questioned (189). It may also have detrimental effects on the welfare of animals. Kentner et al. (190) argues that enrichment improves reproducibility, and Würbel and Garner (191) suggest that it benefits welfare through reducing behavioral pathologies provided the enrichment caters to the biological needs of the species. They argue that in fact systematic environmental randomization could contribute to better science and could in fact be a refinement. The 3 Rs of Russell and Burch include refinement not only in the procedures but in the housing, husbandry, health, and safety of the animals, understanding that endorsing better conditions for animals improves the quality of research (192).

In recent years, the search for objective evaluations of pain has included proposals to use infrared thermography techniques (193), facial electromyography (149), recognition of facial expressions or behavior by means of sensors, automated recognition in production species like sheep (194), artificial vision technology using computers—suggested to recognize pain in horses (195)—and artificial intelligence where machines are taught to recognize certain FAU (196). As these cases reveal, the tendency is to develop precise, non-invasive methods that allow researchers to evaluate pain in laboratory rodents while causing the least stress possible by handling or the simple presence of the evaluator.

## Conclusions

Pain is defined as an unpleasant sensory experience that entails the activation and integration of diverse neurobiological systems that are responsible for transducing and transmitting it and recognizing it consciously. Since laboratory rodents are most often utilized in biomedical science, the recognition of pain constitutes a fundamental step toward complying with existing norms for the use and care of laboratory animals, with the objective of preventing the physiological, endocrine, metabolic, and behavioral consequences described in this review.

The evaluation of pain in laboratory requires understanding the nociceptive pathway and the neurobiology associated with observable changes in facial expressions. Utilization of the FAU described (position of the ears and whiskers, ocular opening, and flattening of the nose or cheeks) has led to the adoption of facial expressions as a non-invasive method for determining degrees of pain (on a scale from 0 to 2) in diverse assays and models of acute, chronic, surgical, and neuropathic pain. The study of facial expressions allows researchers to recognize, objectively and integrally, the presence, degree, and intensity of pain that an animal may experience during its life or euthanasia processes. Thus, it constitutes a complementary tool for refining the use of rodents in research.

## Author contributions

All authors contributed to the conceptualization, writing, reading, and approval of the final manuscript.

## Conflict of interest

The authors declare that the research was conducted in the absence of any commercial or financial relationships that could be construed as a potential conflict of interest.

## Publisher's note

All claims expressed in this article are solely those of the authors and do not necessarily represent those of their affiliated organizations, or those of the publisher, the editors and the reviewers. Any product that may be evaluated in this article, or claim that may be made by its manufacturer, is not guaranteed or endorsed by the publisher.
